# An Evaluation Index System of Basic Elderly Care Services Based on the Perspective of Accessibility

**DOI:** 10.3390/ijerph19074256

**Published:** 2022-04-02

**Authors:** Jinrong Hu, Yuyuan Zhang, Le Wang, Victor Shi

**Affiliations:** 1 School of Public Administration, Xi’an University of Architecture and Technology, Xi’an 710055, China; zhangyuyuanyy@163.com; 2 Xi’an Institute of Space Radio Technology, Xi’an 710100, China; wangle2142@163.com; 3 School of Management, Xi’an Jiaotong University, Xi’an 710049, China; 4 Lazaridis School of Business and Economics, Wilfrid Laurier University, Waterloo, ON N2L 3C5, Canada; cshi@wlu.ca

**Keywords:** accessibility, AHP, basic elderly care services, evaluation index system, population aging

## Abstract

Population aging has become more and more severe in many countries. As a result, the demand for basic elderly care services has risen. The establishment of an evaluation index system for basic elderly care services can provide guidelines for governments to improve the quality of such services. Based on the “5A” theoretical analysis framework of Penchansky and Thomas, this paper introduces the concept of “accessibility” into evaluation. The “accessibility” model of services, through a literature review, field research, and three rounds of expert correspondence, consists of three first-level indicators, including the accessibility of home-based community elderly care services, the accessibility of institutional elderly care services, and the accessibility of administrative services. The evaluation index system of 15 s-level indicators and 70 third-level indicators, using AHP to determine the weight value of each indicator, provides a quantitative basis for the quality evaluation and improvement of basic elderly care services. Based on our quantitative results, policy recommendations are put forward: strengthen the support for the human and financial resources of community home-based elderly care services; improve the affordability of basic elderly care services; increase the types and numbers of institutional elderly care service projects; improve the availability and adaptability of institutional elderly care services; improve the accessibility of administrative services so that elderly care service institutions and elderly care administrative agencies can establish an effective communication and feedback mechanism.

## 1. Introduction

As the global aging trend accelerates, actively coping with population aging has become a national strategy. For example, according to data from the National Bureau of Statistics, in 2020, the proportion of China’s population aged 60 and above will account for 18.7% of the national population, and the proportion of the population aged 65 and above will be 13.5%, an increase of 5.44% and 4.63%, respectively, compared with 2010. Globally, the elderly population is growing at a rate of 2% per year, and the proportion of the elderly population is expected to reach 21% in 2050. Population aging will be one of the major challenges faced by economic and social development at present and in the future [1]. As a basic public service, basic pension can make all the elderly enjoy basic pension services equally and fairly. It lays the foundation for the construction and development of a country’s pension service system and effectively alleviates the pressure brought by population aging [2].

In China, the construction of a three-level pension service system has been the focus of the government’s pension security work, and a basic pension service system “based on home-based pensions, supported by community services and supplemented by institutional pensions”, has been gradually established. However, at this stage, the effective supply of basic old-age services is insufficient, the layout of old-age service facilities does not match the spatial distribution of the elderly population, the low-paid and unpaid services provided by the government are limited, and the elderly cannot afford old-age services. There are still problems, such as considerable differences in the basic elderly care services, and there is still a lack of systematic and standardized evaluation standards and evaluation systems for basic elderly care services.

Therefore, establishing and improving the evaluation index system of basic elderly care services and comparing and evaluating its quality in the same region are key measures to improve its quality and achieve the “inclusive” elderly care policy. This paper introduces the concept of “accessibility” in the field of health services into the evaluation process of basic elderly care services in districts and counties. In each aspect, the economic, geographical, and service quality of basic elderly care services are inspected. An evaluation index system for basic elderly care services is proposed. The aim of this paper is to further promote the development of the elderly care industry and provide managerial and policy insights for improving its quality.

## 2. Literature Review

In the evaluation of elderly care services, measurement debriefing system (MDS) quality evaluation system and service quality scale are often widely used. The MDS quality evaluation system released by the American Medical Research Association in the 1990s included 175 indicators, which were widely used in the evaluation of service quality in elderly care institutions. However, MDS is mainly graded according to the functional status and health of the elderly and has a certain degree of subjectivity in the weight of life support and medical needs, which is not convincing [3]. In addition, the SERVQUAL scale, which evaluates elderly care services according to the gap between the service quality expected by customers and the actual perceived service quality, is also widely used. At present, relevant research on the quality evaluation of elderly care services at home and abroad mainly focuses on two aspects.

The first is the evaluation of the service quality of elderly care institutions. (1) From the research on service evaluation content of elderly care institutions, Wadhera et al. (2020) developed QMS based on the MDS quality evaluation index system, which consists of 14 indicators, covering psychological characteristics, clinical complexity, elderly function status, etc. [4]. Zhang et al. (2019) evaluated the organizational management and resources, service content and methods, service feedback, and rights and interests in the elderly care institutions by constructing a three-dimensional theoretical framework for the evaluation of service quality in elderly care institutions [5]. Favez et al. (2020) constructed six quality indicators of elderly care institutions covering four clinical areas, and these indicators can compare the differences in the level of nursing quality among various elderly care institutions [6]. (2) From the research on service evaluation methods in nursing homes, Chamberlain et al. (2017) used MDS2.0 to collect data on nursing homes, and the results showed that MDS could help improve the health and quality of life of the elderly [7]. Yuan et al. (2020) introduced the three-stage DEA model into the field of efficiency evaluation of the combination of medical and nursing care. They conducted a three-stage DEA model analysis on 53 integrated medical and nursing services in Tianjin [8].

The second is the evaluation of community home-based care services. (1) In evaluating community home-based care services, Vestjens L et al. (2016) evaluated community elderly care services through the evaluation of the physical condition of the elderly, the establishment of case management, an individualized comprehensive primary care system, a social coordination team, self-care of the elderly, and shared care [9]. Zhu et al. (2019) established a service quality evaluation index system for community home-based elderly care centers with service structure quality, service process quality, and service result quality as the first-level indicators [10]. (2) For the research on the evaluation method of community home-based elderly care services, Song et al. (2014) used the SERVQUAL model to construct and analyze the urban community elderly care service model and put forward suggestions for the quality, status, and development of community elderly care services [11]. (3) For the research on the accessibility of community home-based care services, Yong et al. (2018) used the fuzzy comprehensive evaluation method to conduct a comprehensive evaluation of the accessibility of community services in Beijing, Nanjing, and Xianyang by building a conceptual model of the accessibility of home-based care community services [12]. Comprehensive evaluation by Di et al. (2020) introduced the sustainability of medical services based on the accessibility theory. They analyzed the medical services for the elderly at home in the Shaanxi Province through three aspects: potential accessibility, achievable accessibility, and sustainable accessibility of medical services. The total accessibility and sustainable accessibility were evaluated [13].

To sum up, scholars mainly conduct research on institutional elderly care services or community home-based elderly care services from the perspective of evaluation content and methods. However, there is little research on the multi-level and multi-dimensional comprehensive evaluation of basic elderly care services. For the research on the accessibility of elderly care services, most of them focus on the accessibility of home-based elderly care services in the community, and little research has been conducted on the accessibility of elderly care services in institutions. However, taking districts and counties as the research object, combining the accessibility of community elderly care services with the accessibility of institutional elderly care services, and considering the accessibility of administrative services, there is no scholarship involved. Therefore, based on the actual needs of local governments, this research’s key point is to build a comprehensive evaluation index system for appropriate basic elderly care services. By sorting out relevant literature on accessibility, this paper constructs a comprehensive conceptual model of basic elderly care service accessibility covering home-based community elderly care, institutional elderly care, and administrative services, and then establishes an evaluation index system for basic elderly care services in districts and counties, striving to be comprehensive and objective. The evaluation of the basic elderly care service level in districts and counties provides a quantitative basis for evaluating the quality of basic elderly care services in districts and counties.

## 3. Construction Basis of Evaluation Index System of Pension Services

### 3.1. Concepts and Models of Accessibility

Accessibility was first used in the field of health services, which was mainly used in the quality, efficiency, and fairness evaluation of health service systems. On accessibility, there are two classic studies. From the perspective of fit, Campbell argues “accessibility is the availability of services, that is, whether the service object can obtain convenient, timely, affordable and acceptable services” [14]. Penchansky and Thomas (1984) put forward the concept of “accessibility” for customer and customer service system adaptation. In addition, “accessibility five-dimensional measurement method” is constructed, which evaluates accessibility from five aspects: availability, accessibility, affordability, suitability, and acceptability [15]. Table 1 lists their specific meanings. From the perspective of “service use,” Andersen (1995) defines accessibility as the actual use of health services by individuals and various factors that promote and hinder the use of services, and divides “accessibility” into potential accessibility, realization accessibility, fair accessibility, unfair accessibility, and effective accessibility for evaluation [16]. Ma (2020) defines “accessibility” as a function of the service provider, mainly the degree to which the service system is open to and meets the needs of the target population, which affects the successful entry of individuals with service needs, i.e., the ability to use and exit appropriate services [17]. Wang et al. (2017) believe that accessibility has the characteristics of convenience, acceptability, and affordability, and accessibility is mainly reflected in the matching degree of “demand” and “supply” in rural basic pension services [18].

The meaning of “accessibility” is very complicated. This paper constructs the evaluation index system of basic old-age care services in districts and counties from the perspective of “suitability”. Combined with Penchansky and Thomas’ “5A” analysis framework of health service accessibility, usability, accessibility, affordability, adaptability, and acceptability are applied to the construction of evaluation index system of basic old-age care services in districts and counties, and its contents are revised and extended. Table 2 shows the conceptual model for evaluating the accessibility of basic pension services in districts and counties. By analyzing the accessibility of basic pension services in districts and counties, it provides insights for evaluating the development level and service quality of basic pension services in districts and counties.

### 3.2. The Connotation of Basic Pension Services

Establishing and improving the basic old-age service system and better meeting the diversified old-age needs of most elderly is an important measure to improve a country’s old-age service system. In China, it was clearly proposed for the first time that the basic old-age service system should be improved so that the basic old-age service can play an essential and guaranteed role in “supporting the elderly”. In 2021, the “National Basic Public Service Standards” in China clearly stipulated the scope and quality of basic public services for the elderly from the aspects of health management of the elderly, welfare subsidies for the elderly, basic pension insurance for employees, and basic pension insurance for urban and rural residents.

There is still debate in the academic community about the definition of the connotation of basic old-age services. Some scholars have tried to define the connotation of basic old-age services. Luan et al. (2013) believed that basic pension services mainly refer to a network composed of facilities, organizations, talents, and technical elements, such as basic life care, nursing and rehabilitation, mental care, emergency rescue, and social participation, which is adapted to the level of economic development and aims to meet the basic service needs of the elderly and improve the quality of life of the elderly [19]. Li et al. (2015) believe that the basic old-age service refers to the government for those who cannot rely on their own pension services, which must, therefore, be provided through government assistance to the elderly and their families, through public financial investment, facilities construction, service supply, and supervision and management of a series of policies and practical activities; it must provide basic old-age consultation, care, rehabilitation, and mental health services [20]. Huang et al. (2020) proposed that the basic pension service is dominated by the government, and enterprises, social organizations, and other departments are involved in the implementation. The service objects are all the elderly aged 60 and above. The basic pension service is provided through public financial investment for the elderly and their families who rely on themselves to obtain pension services and must seek help from the government [21].

In summary, most scholars define the object of basic old-age care services as the elderly with special difficulties. Still, from the definition of pension policy texts in recent years, the object of basic old-age care services has changed, from the elderly with special difficulties to the broader elderly. Therefore, based on the research of scholars, the change of government policy, and the practical needs of old-age care, this paper defines the basic old-age care service as dominated by the government and focuses on the special elderly groups, such as disability, poverty, and “three-no”, to protect the basic survival and development rights of all the elderly, meet the general basic old-age needs of all the elderly, and ensure that all the elderly can have equal opportunities, fair and timely access to old-age services to meet the basic needs of life.

## 4. Construction of the Evaluation Index System of District and County Elderly Care Services

### 4.1. Research Methods

According to the content characteristics of pension services, this paper divides the basic pension services in districts and counties into home-based community pension services and institutional pension services. Combined with the administrative management characteristics of pension services in districts and counties, the pension administrative services are included in the first-level indicator system. According to the “5A” analysis framework of Penchansky and Thomas’ accessibility, the evaluation index system of basic old-age care services is preliminarily constructed. Next, the index system is supplemented and revised according to the expert advice and results, and then, the index weight is determined by the analytic hierarchy process. Finally, a perfect evaluation index system of basic old-age care services in districts and counties is obtained.

The article refers to the relevant laws and regulations and policy texts in the preliminary index system construction. It includes the Measures for the Administration of Pension Institutions issued by the Ministry of Civil Affairs in 2020 (Decree No. 66 of the Ministry of Civil Affairs) [22], the Law of the People’s Republic of China on the Protection of the Rights and Interests of the Elderly [23], the Basic Standards for the Service Quality of Pension Institutions (GB/T35796—2017) [24], the Basic Requirements for the Service of Community Day Care Centers for the Elderly (GB/T33168—2016) [25], the Opinions on Promoting the Development of Pension Services (No. 5 of the State-Office) [26], the Customer Satisfaction Assessment of Pension Institutions (MZ/T133—2019) [27], and the 14th Five-Year Plan. Concerning the research on the evaluation system of home care services, community care services, and institutional care services, the indicators are selected, modified, and adjusted to ensure the accuracy and effectiveness of the indicators.

After preliminarily determining the evaluation index system of district and county pension services through literature research, the Delphi method is used to optimize the evaluation index system further, and a scientific and reasonable evaluation index system is obtained. The specific steps are as follows. First, questionnaires are distributed in the form of face-to-face distribution and online distribution. Second, expert consultations are conducted. This paper has three rounds of expert consultation. In the first round of expert consultations, experts used the Likert5 grading method to judge and score the importance of each indicator and set up an opinion modification column. In the second round of expert consultation, the first round of consultation results is provided to experts anonymously, and the index weight assignment column is set in the consultation table. At the same time, experts judge, evaluate, and score the modified evaluation index system. In the third round of expert consultation, some experts scored the weight of the index system and used the analytic hierarchy process (AHP) to score the weight of the formed evaluation index system to determine the weight value of each index.

### 4.2. Index Screening and Result Statistics

After the questionnaire was collected, the questionnaire was collated and analyzed, and the average, standard deviation, and coefficient of variation (CV) were calculated according to the importance of each index. The standard of index screening was that the average score of importance was >3.50, and the coefficient of variation was <0.25. At the same time, combined with expert advice, the indicators were screened to remove indicators that did not meet the standard.

Excel 2021 and SPSS25.0 were used to analyze the data. The expert consultation reliability was analyzed by the expert positive coefficient, expert opinion authority, and expert opinion coordination. The expert positive coefficient is represented by the questionnaire recovery rate; the authority coefficient (Cr) of indicators is used to indicate the authority of expert opinion; Kendall coordination coefficient (W) and coefficient of variation (CV) are used to represent the coordination degree of expert opinions. The larger the value of W is, the better the coordination degree of experts is. Experts on the evaluation of indicators used the importance score mean, coefficient of variation (CV) and full score rate, and index weight.

## 5. Analysis of the Results of the Evaluation Indicators of the Old-Age Care Services in Districts and Counties

### 5.1. Basic Situation of Experts

**Figure 1:**
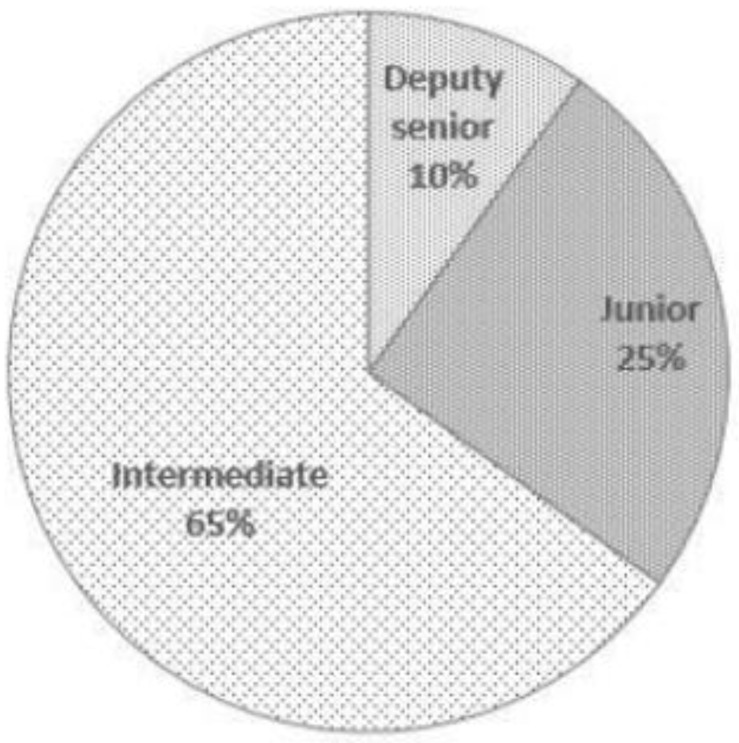
Professional titles of experts.

**Figure 2:**
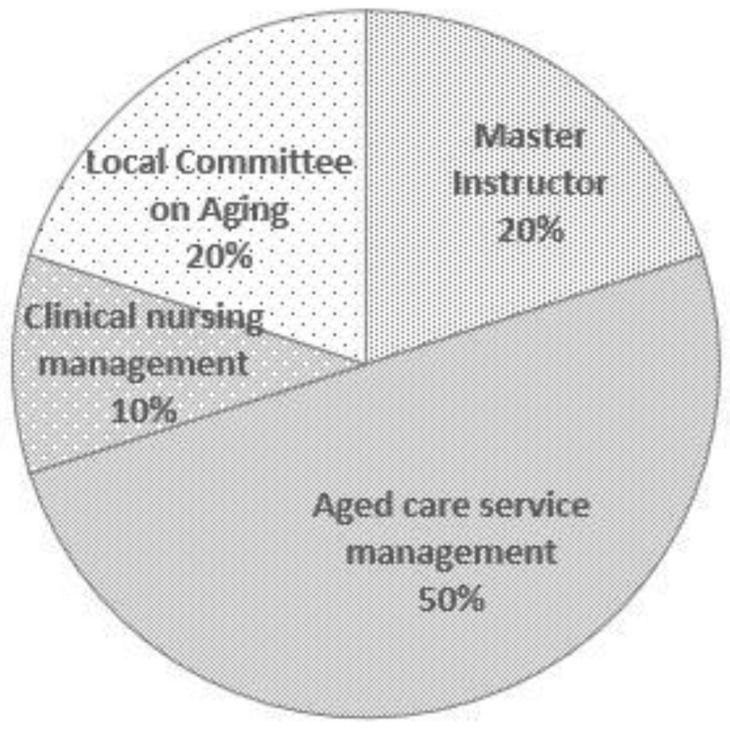
Expert areas of expertise.

There are 20 experts in this study. Table 3, Figure 1 and Figure 2 are the details of the experts participating in this study. The experts participating in this consultation have a solid theoretical basis and sufficient practical experience in pension services.

This expert consultation requires experts to be engaged in the management of elderly care services for more than five years, or experts in the social welfare industry and clinical nursing management with sub-senior technical titles or above; to be familiar with the construction of the basic elderly care system and the research and development of elderly care management; to be able to guarantee participation in at least two rounds of expert consultation.

### 5.2. Analysis of Expert Participation

#### 5.2.1. The Enthusiasm of Experts and the Degree of Authority of Experts

In the first and second rounds of expert consultation, 20 questionnaires were distributed and 20 were recovered, with a recovery rate of 100%. The authority degree of expert opinion (Cr) is calculated from the degree of familiarity (Ca) and the basis for judging indicators (Cs), that is, Cr = (Cs + Ca)/2. Table 4 shows the Cs, Ca, and Cr values of the first and second rounds of expert inquiries. The expert authority coefficient (Cr) of both rounds was >0.800, indicating that the expert authority was high [28].

#### 5.2.2. Degree of Expert Coordination

The coefficients of variation (CV) of the first and second rounds of expert correspondence were 0.07 to 0.23, 0 to 0.23 (CV < 0.25); the expert coordination coefficients of the first and second rounds of expert correspondence (Kendall’s W) were 0.192 and 0.196, respectively, and the W value test of the two rounds of expert correspondence was statistically significant (both *p* < 0.01).

### 5.3. Analysis of the Inquiries of Indicators

According to the index screening conditions and expert opinions, 1 first-level indicator, 5 s-level indicators, and 22 third-level indicators were added, and the “number of nursing beds per thousand elderly people” in the third-level indicator was revised to “the number of community pension service beds in the administrative area”; modify “the number of nursing staff in household/nursing elderly care services” to “the ratio of the number of nursing staff in household/nursing elderly care services to the number of elderly people receiving services”; modify “every thousand the number of institutional pension beds owned by the elderly” to “the number of beds in the elderly care institutions in the administrative area”; “acceptance of the environment in which the elderly care services are located” to “satisfaction with the surrounding environment where the nursing care services provided by institutions are located”; increase the indicators “satisfaction with the environment in which the community-provided care and elderly care services are located” and “satisfaction with the hardware facilities of the care and elderly care services provided by institutions”. According to expert opinions, the details of some indicators were revised, and finally, the evaluation indicator system for basic elderly care services in districts and counties was drawn up.

According to the consistency test results of expert evaluation, the consistency ratios (CR) of this study are all ≤0.1, indicating that the judgment matrix of this study satisfies the consistency test, and the calculated weights satisfy the consistency. Therefore, a district/county basic elderly care service evaluation index system, including 3 first-level indicators, 12 second-level indicators and 69 third-level indicators has been established. (See Appendix A for details)

### 5.4. Analysis of the Results of the Index Inquiry

#### 5.4.1. Inquiry Results of the First-Level Indicators

The weights of the first-level indicators are ranked in descending order: the accessibility of institutional elderly care services (0.429), the accessibility of administrative services (0.428), and the accessibility of home-based community elderly care services (0.143). The results are shown in Table 5. The accessibility of institutional elderly care services accounts for a large proportion. Using expert interviews, this result is due to the current shortage of elderly care institutions and the lack of beds. At the same time, the service quality of elderly care institutions is uneven, and the supply and demand of older adults staying in elderly care institutions are misaligned; the accessibility of administrative services is second only to institutional elderly care services. The conclusion from this is that there are few evaluation studies on pension administrative services. Most evaluation systems tend to ignore the problems of pension administration, and pension administration services play a guiding, commanding, and supervising role in the process of pension services. According to the results of research and interviews, it is found that the main reason is that, as an important part of the three-tier pension system, community home-based pension services are the fastest growing among the three pension models. Still, the development of their service model is not yet mature. Compared with institutional elderly care, due to the large proportion of the elderly who can take care of themselves among the elderly at home, limited by their consumption concepts, the demand for home elderly care services is currently smaller than that for institutional elderly care (the main occupants are the demand for services for the disabled and semi-disabled elderly), making the availability of home-based and community-based elderly care services less urgent at this stage. However, as the proportion of the elderly population increases, the demand for home-based care services will also increase, and the accessibility of home-based community care services will also increase in the future.

#### 5.4.2. Inquiry Results of the Second- and Third-Level Indicators

The indicators with serial numbers A1–A5 are the sub-indices of the accessibility of home-based and community-based elderly care services, and A11–A57 are the three-level indicators of the accessibility of home-based and community-based elderly care services. Table 6 shows the results of the second-level and third-level indicators of the accessibility of home-based and community-based elderly care services, affordability (0.116), acceptability (0.115), and adaptability (0.116). The survey results show that from the point of view of availability, the resources of home-based and community-based elderly care services are insufficient at this stage. The types of services are relatively single, mainly for the elderly, and do not yet fully cover the urban elderly population. The basic old-age service resources provided are not enough to meet the old-age needs of the elderly. From the perspective of accessibility, due to the inconvenience of the elderly, the nearby elderly care is one of the means to improve the experience of elderly care services, but due to the constraints of human, material, and financial resources, the current community home-based elderly care service facilities do not yet achieve full coverage. Living care services belong to the basic services in community home-based elderly care services, so living care services account for a large proportion of affordability. In terms of adaptability, the elderly have a relatively great demand for medical care services due to the decline of their physical functions. Therefore, the most important factor in evaluating the satisfaction of community-provided elderly care services is the elderly’s satisfaction with medical care services.

B1–B5 are the second-level indicators of the accessibility of institutional elderly care services, and B11–B58 are the third-level indicators of the accessibility of institutional elderly care services. Table 7 shows the inquiry results of the second- and third-level indicators of the accessibility of institutional elderly care services. Affordability (0.087), affordability (0.086), acceptability (0.086). For the availability of institutional elderly care services, the number of elderly service resources has an equally important role in the quality of elderly care services. The number of institutional nursing staff is an important guarantee for the quality of elderly care services. In terms of affordability, the cost of institutional pensions is relatively high, and most older adults have low-income levels, so the total fees of pension institutions account for a larger proportion of affordability. In terms of acceptability, some older adults still have a low degree of acceptance of the model and content of institutional pensions. Therefore, the two account for the same proportion in terms of the acceptability of institutional pensions. In institutional elderly care services, the elderly’s satisfaction with life care services and medical care services is equally important. This is because most elderly who choose institutional care for the elderly are disabled and semi-disabled. Additionally, the demand for medical care services is higher than the active elderly or healthy elderly.

The serial numbers C1–C5 are the second-level indicators of the accessibility of administrative services, and C11–C55 are the third-level indicators of the accessibility of administrative services. Table 8 shows the results of the second- and third-level indicators of the accessibility of administrative services. For the accessibility of administrative services, the availability of administrative service resources accounts for the most significant proportion (0.527), which is due to the elderly care services provided by administrative services. Resources are critical to the provision of aged care services. The satisfaction of each stakeholder with the work of the administrative department is also an important part in judging the level of elderly care services in districts and counties. For the availability of administrative services, the ratio of service managers in elderly care institutions to the number of older adults receiving services is an essential basis for judging the availability of administrative services. Therefore, it is necessary to focus on the number of managers and the proportion of the elderly. The coverage rate of elderly service centers is an important indicator that affects the accessibility of administrative services. As the number of elderly populations increases year by year, the demand for elderly service centers is also gradually increasing. In terms of affordability, the important function of basic old-age care services is to provide basic guarantees for the elderly in difficulty, so the proportion of older adults who receive old-age support subsidies in the administrative region is more important. In terms of acceptability, streamlining administration and delegating power to administrative departments can reduce the burden on elderly care institutions. In terms of adaptability, pension policies have a direct impact on pension institutions and home-based pension service stations, so satisfaction with pension policies is an important basis for judging the availability of administrative services in districts and counties.

## 6. Discussion

### 6.1. Reliability of Letter Inquiry Results

Both rounds of expert letter inquiries were 100% recovered, and the recovery rate was ≥70%, indicating that the results of the letter inquiries were good [29]. In addition, the 20 experts selected in this study have rich working experience in elderly care institution management and clinical nursing, and the authoritative coefficients of the two rounds of expert correspondence are >0.800, indicating that the experts participating in this research have a high degree of overall authority. This ensures the reliability of the content of the inquiry.

### 6.2. Characteristics of the Indicator System

#### 6.2.1. The Scientific Nature of the Indicator System

China’s old-age service has formed a multi-level old-age service system based on “home-based, community-based and institution-based” care. Based on this, this study selects home-based community old-age service and institutional old-age service as first-level indicators. On this basis, combined with the reality, considering the current pension service development process, the pension service resources provided by administrative services have a crucial impact on pension services, so the administrative services are included in the first-level indicators. Combined with the Penchansky and Thomas health service accessibility index system, three first-level indicators of home-based community care service accessibility, institutional care service accessibility, and administrative service accessibility are constructed according to the different pension modes. The secondary index is a further extension of the connotation of Penchansky and Thomas’ evaluation model. The primary index is decomposed into five dimensions: availability, accessibility, affordability, acceptability, and accessibility, to measure the primary index concretely. The third-level indicator is a deep analysis of the content of the second-level indicator. Through the objective, comprehensive and scientific evaluation of the content and form of basic pension services in districts and counties, the evaluation content is refined, and the operability of the indicator system is strengthened. Through the above three levels of indicators, the evaluation index system of basic pension services in districts and counties is constructed to measure the basic pension services in an all-round and multi-level way and further promote the improvement and development of basic pension services in China.

#### 6.2.2. Comprehensiveness of Evaluation Content

Through the construction of three major index systems: the accessibility of home-based elderly care services, the accessibility of institutional elderly care services, and the accessibility of administrative services, the above indicators are further subdivided into availability, accessibility, affordability, acceptability, and accessibility. The five aspects of adaptability involve many aspects of basic old-age services, including the resource conditions, convenience, the reasonableness of service prices, acceptance of old-age methods, satisfaction of old-age services, and old-age administration. This study not only includes some basic old-age service evaluation content but also adds the old-age administrative evaluation content on the original basis. The old-age administrative service has an important impact on the level of the old-age service and influences the level of the old-age service. The index system of this study evaluates the basic elderly care service level of districts and counties from multiple perspectives, comprehensively and at multiple levels, and effectively evaluates the level of elderly care services in districts and counties, to provide good elderly care services for the elderly.

### 6.3. Limitations

This study aims to take advantage of expert advice to establish the evaluation index system of basic pension services in districts and counties, which is of great significance to standardize the level of basic pension services and improve the quality of basic pension services. However, at present, the evaluation of basic pension services in districts and counties in China is still at an early stage. Additionally, future research can be conducted to build such systems in other countries and compare the systems for different countries. Moreover, there is no mature evaluation criterion. Secondly, the index system proposed in this study is currently only a framework, and specific evaluation scales need to be developed in the future to better evaluate the basic pension service level in districts and counties.

### 6.4. Prospect

This paper constructs a comprehensive evaluation index system of basic pension services in districts and counties, which provides a basis for the objective evaluation of the quality of basic pension services. In the follow-up study on the evaluation of basic pension services, we will take a city as an example for empirical research. By collecting the relevant data of the counties in this area, this evaluation index system is used to measure the basic pension service level. Policy incentives are carried out for the counties with better evaluation results. Further guidance is provided for the counties with poor evaluation results to promote the development and improvement of the basic pension service level of the counties in China.

## 7. Conclusions 

Population aging is one of the significant challenges that China needs to face in the future. The effective measure to deal with the problem of population aging is to build a forward-looking evaluation standard to promote the rapid development of the pension industry actively. At present, the development of basic pension service evaluation in districts and counties in China is not yet mature, and the research on evaluation index system is relatively lacking. Based on the evaluation of the basic pension service level in districts and counties, this study establishes a practical evaluation index system for the basic pension service by selecting experts from relevant parties through consultation. According to the calculated index weight results and expert advice, the county can improve the quality of basic old-age care services from the following aspects: First, to enhance the availability of home community old-age care services, increase the type and quantity of care / home care services, increase the support of human, material, and financial resources of community old-age care service stations, and complement the shortcomings of community home care services to meet the needs of the elderly; second, strengthen the accessibility of institutional pension services, especially the types of pension services provided by institutions, increase the number of institutional nursing staff, strengthen the talent reserve construction of pension service teams, improve the adaptability of institutional pension services, and improve the satisfaction of the elderly with the life care services and medical care services provided by institutions; third, improve the accessibility of pension administrative services, especially in the availability and acceptability of administrative services, so that pension service institutions and pension administrative departments can establish effective communication, establish effective feedback mechanism, and form a virtuous circle.

In this paper, the evaluation index system of basic old-age care services based on the Delphi method is of great significance to standardize the level of basic old-age care services in districts and counties and improve the quality of basic old-age care services. At the same time, it also provides insights for the construction of other evaluation index systems of old-age care services. With more empirical research in the future, it can improve the level and quality of basic pension services and help guide the evaluation of basic pension services.

## Figures and Tables

**Table 1:** Penchansky and Thomas’ five-dimensional measurement.

Concept Name	Definition
Availability	Relationship between supply side and target group needs in terms of resources, number, and type of services
Accessibility	The convenience of geographic space of service resources for target groups
Accountability	The acceptance of service price by target groups
Accommodation	Whether the target group recognizes the way suppliers provide services
Acceptability	Relationship between service provider characteristics and target group characteristics

**Table 2:** Conceptual model of accessibility of basic pension services.

Indicator	Definition	Contents	Breakdown
Availability	The relationship between basic old-age service resources and old-age service resources needed by the elderly	The ability of district and county institutions and communities to provide basic elderly care service resources	Aged care service items/types
Accessibility	The degree of matching between the distribution of the elderly population and the geographical location of elderly care facilities	The distance between the residence of the elderly and the elderly care institutions and the degree of institutional coverage; the degree of matching between the distribution of the elderly population and the geographical location of service facilities	Distance to home from aged care service station/aged care institution
Affordability	Older people’s acceptance of elderly care service prices	The affordability of the various elderly care services to the elderly	Affordability of various aged care services
Acceptability	The evaluation results of the elderly on the quality of elderly care services	The degree of recognition of the elderly on the type, content, and form of elderly care services	Acceptance of the content or model of elderly care services
Adaptability	The elderly’s acceptance of the content or form of elderly care services	Older people’s satisfaction with elderly care services	Satisfaction with various elderly care services

**Table 3:** Basic information of experts.

Item		Number of People
Age	28–40	3
	41–50	13
	51–60	4
Working years	5–10	2
	10–20	15
	20 or more	3
Region	East China South China North China Central China Northeast Southwest Northwest	3 3 2 3 2 3 4

**Table 4:** Statistical table of authority degree of experts.

Project	Judgment Basis (Cs)	Familiarity (Ca)	Authority (Cr)
The first round of expert correspondence	0.86	0.82	0.84
The second round of expert correspondence	0.88	0.82	0.85

**Table 5:** Inquiry results of the first-level indicators of the indicator system.

Index	Importance Assignment (X ± S)	Importance Assignment (X ± S)	Coefficient of Variation (CV)	Coefficient of Variation (CV)
A. Availability of home-based community aged care services	5.00 ± 0.00	1.00	0.00	0.143
B. Institutional aged care service accessibility	5.00 ± 0.00	1.00	0.00	0.429
C. Administrative services accessibility	5.00 ± 0.00	1.00	0.00	0.428

**Table 6:** Availability of home-based and community-based elderly care services.

Index	Importance Assignment	Full Score	Coefficient of Variation (CV)	Index Weight
A1 Availability	4.15 ± 0.36	0.15	0.087	0.523
A11 Types of nursing/home-based elderly care services provided by the community	4.15 ± 0.48	0.20	0.12	0.383
A12 Number of childcare/home-based elderly care services provided by the community	4.20 ± 0.51	0.25	0.23	0.138
A13 The ratio of the number of nursing staff in household/nursing care services to the number of elderly people receiving services	4.15 ± 0.48	0.20	0.12	0.094
A14 Number of equipment and facilities in community elderly care services	4.20 ± 0.51	0.25	0.12	0.101
A15 The number of community elderly care service sites in the administrative area	4.45 ± 0.50	0.45	0.11	0.094
A16 Percentage of certified nursing staff in household/nursing care services	4.30 ± 0.56	0.35	0.13	0.101
A17 Number of beds for home-based care services in the administrative area	4.30 ± 0.46	0.30	0.11	0.090
A2 Accessibility	4.30 ± 0.46	0.30	0.11	0.130
A21 The distance from the residence of the elderly to the home-based elderly care service station	4.15 ± 0.36	0.15	0.09	0.429
A22 Convenience of the elderly’s residence to the home-based elderly care service station	4.20 ± 0.40	0.20	0.10	0.428
A23 Accuracy rate of nursing staff’s home service time	4.30 ± 0.56	0.35	0.13	0.143
A3 Affordability	4.30 ± 0.46	0.30	0.11	0.116
A31 Affordability of access to living care services	4.40 ± 0.58	0.45	0.13	0.465
A32 Affordability of access to health care services	4.40 ± 0.49	0.4	0.11	0.309
A33 Affordability of access to health management services	4.25 ± 0.43	0.25	0.10	0.075
A34 Affordability of access to spiritual comfort services	4.30 ± 0.46	0.30	0.11	0.076
A35 Affordability of access to cultural and recreational services	4.40 ± 0.49	0.40	0.11	0.076
A4 Acceptability	4.25 ± 0.43	0.25	0.10	0.115
A41 Acceptance of the content of household/nursing care services	4.50 ± 0.59	0.55	0.13	0.500
A42 Acceptance of the household/nursing care model	4.50 ± 0.59	0.55	0.13	0.500
A5 Adaptability	4.20 ± 0.40	0.20	0.10	0.116
A51 Satisfaction with life care services provided by the community	4.50 ± 0.50	0.50	0.11	0.137
A52 Satisfaction with health care services provided by the community	4.50 ± 0.50	0.50	0.11	0.201
A53 Satisfaction with health management services provided by the community	4.55 ± 0.50	0.55	0.11	0.091
A54 Satisfaction with spiritual comfort services provided by the community	4.50 ± 0.50	0.50	0.11	0.088
A55 Satisfaction with cultural and recreational services provided by the community	4.50 ± 0.50	0.50	0.11	0.172
A56 Satisfaction with the quality of elderly care services provided by the community	4.30 ± 0.46	0.30	0.11	0.143
A57 Satisfaction with the environment in which the community-provided nursing care services are located	4.40 ± 0.49	0.40	0.11	0.168

**Table 7:** Institutional aged care service accessibility.

Index	Importance Assignment	Full Score	Coefficient of Variation (CV)	Index Weight
B1 Availability	4.55 ± 0.50	0.55	0.11	0.527
B11 Types of aged care services provided by institutions	4.60 ± 0.49	0.60	0.11	0.097
B12 Number of elderly care services provided by institutions	4.20 ± 0.68	0.40	0.11	0.094
B13 Number of facilities for elderly care services in institutions	4.25 ± 0.70	0.30	0.16	0.161
B14 Ratio of nursing staff in institutions to seniors served	4.30 ± 0.71	0.35	0.16	0.281
B15 Number of elderly care institutions in the administrative area of the district	4.35 ± 0.73	0.50	0.17	0.097
B16 Credentialing rate of institutional nursing staff	4.35 ± 0.57	0.40	0.13	0.135
B17 Number of beds in elderly care institutions in the administrative region	4.25 ± 0.54	0.30	0.13	0.135
B2 Accessibility	4.40 ± 0.49	0.40	0.11	0.087
B21 The distance between the residence of the elderly and the nursing facility	4.35 ± 0.48	0.35	0.11	0.833
B22 Convenience of the elderly’s residence to nearby elderly care institutions	4.35 ± 0.48	0.35	0.11	0.167
B3 Affordability	4.50 ± 0.50	0.50	0.11	0.086
B31 Affordability of comprehensive charges for aged care institutions	4.60 ± 0.49	0.60	0.11	0.350
B32 Affordability of access to living care services	4.30 ± 0.46	0.30	0.11	0.196
B33 Affordability of access to health care services	4.20 ± 0.68	0.35	0.16	0.220
B34 Affordability of access to health management services	4.30 ± 0.71	0.45	0.17	0.079
B35 Affordability of access to spiritual comfort services	4.30 ± 0.46	0.30	0.11	0.079
B36 Affordability of access to cultural and recreational services	4.50 ± 0.50	0.50	0.11	0.076
B4 Acceptability	4.50 ± 0.50	0.50	0.11	0.086
B41 Acceptance of the content of elderly care services provided by institutions	4.55 ± 0.50	0.55	0.11	0.500
B42 Acceptance of institutional pension model	4.45 ± 0.50	0.45	0.11	0.500
B5 Adaptability	4.35 ± 0.48	0.35	0.22	0.214
B51 Satisfaction with living care services provided by the institution	4.45 ± 0.50	0.45	0.11	0.231
B52 Satisfaction with the health care services provided by the institution	4.50 ± 0.50	0.50	0.11	0.231
B53 Satisfaction with the health management services provided by the institution	4.40 ± 0.49	0.40	0.11	0.053
B54 Satisfaction with spiritual comfort services provided by the institution	4.60 ± 0.49	0.60	0.10	0.053
B55 Satisfaction with the cultural and recreational services provided by the institution	4.60 ± 0.49	0.60	0.10	0.053
B56 Satisfaction with the quality of elderly care services provided by institutions	4.55 ± 0.50	0.55	0.11	0.160
B57 Satisfaction with the surrounding environment where the nursing care services provided by the institution are located	4.20 ± 0.87	0.35	0.21	0.110
B58 Satisfaction with the hardware facilities of nursing care services provided by institutions	4.60 ± 0.49	0.60	0.11	0.110

**Table 8:** Access to administrative services.

Index	Importance Assignment	Full Score	Coefficient of Variation (CV)	Index Weight
C1 Availability	4.30 ± 0.46	0.30	0.11	0.372
C11 The ratio of pension administrators to the elderly population in the administrative area	4.25 ± 0.43	0.25	0.10	0.156
C12 The ratio of service managers in elderly care institutions to the number of elderly people receiving services	4.35 ± 0.48	0.35	0.11	0.311
C13 The ratio of public pension beds to the number of elderly people in the administrative area	4.15 ± 0.36	0.15	0.09	0.104
C14 Average waiting time for admission in public aged care institutions	4.15 ± 0.36	0.15	0.09	0.104
C15 The employment rate of senior care administrators in this administrative area with certificates	4.25 ± 0.43	0.25	0.10	0.104
C16 The intensity of information disclosure of elderly care services within the administrative region	4.05 ± 0.59	0.15	0.15	0.222
C2 Reachability	4.25 ± 0.43	0.25	0.10	0.064
C21 Coverage rate of each sub-district and each community elderly care service center (home elderly care service station, day care center, etc.)	4.00 ± 0.55	0.10	0.14	0.750
C22 Coverage of elderly care institutions near the place where the elderly live	4.00 ± 0.32	0.05	0.08	0.250
C3 Affordability	4.30 ± 0.46	0.30	0.11	0.172
C31 Proportion of the number of elderly people receiving pension support	3.85 ± 0.48	0.05	0.12	0.442
C32 The total amount of support subsidies for various elderly care services in the administrative region	3.95 ± 0.50	0.10	0.16	0.234
C33 The total amount of subsidy received by elderly care institutions in the administrative region	3.95 ± 0.50	0.10	0.13	0.168
C34 The total amount of support subsidies received by home-based elderly care service stations in the administrative region (in the past 3 years, including construction subsidies and operating subsidies)	4.05 ± 0.22	0.05	0.05	0.155
C4 Acceptability	4.50 ± 0.50	0.50	0.11	0.219
C41 The degree of acceptance of elderly care institutions/home-based elderly care service stations to the administrative functions of the relevant administrative departments	4.10 ± 0.30	0.10	0.07	0.325
C42 The degree of acceptance of the supervision and management functions of relevant administrative departments by elderly care institutions/home-based elderly care service stations	3.95 ± 0.38	0.05	0.10	0.192
C43 The degree of acceptance of the government’s administrative functions to accurately promote the development of elderly care services by elderly care institutions/home-based elderly care service stations	3.90 ± 0.54	0.05	0.12	0.242
C44 The degree of acceptance of elderly care institutions/home-based elderly care service stations to the administrative functions of relevant administrative departments to optimize the market environment	4.00 ± 0.45	0.10	0.11	0.242
C5 Adaptability	4.25 ± 0.54	0.30	0.13	0.173
C51 Old-age care institutions/home-based care service stations for the supervision of old-age services in the administrative region	4.15 ± 0.36	0.15	0.09	0.123
C52 Residents’ satisfaction with the elderly care services in the district and county	4.15 ± 0.36	0.15	0.09	0.233
C53 Residents’ satisfaction with the administrative department’s old-age service guarantee work	4.15 ± 0.36	0.15	0.09	0.189
C54 Satisfaction of residents with pension policies formulated by administrative departments	4.10 ± 0.30	0.10	0.07	0.341
C55 Residents’ satisfaction with the work of home-based renovation (indoor) within the administrative area	4.15 ± 0.36	0.15	0.09	0.114

## Appendix A

**Table A1:** Evaluation Index System for Basic Old-Age Care Services in Districts and Counties.

Index	Weight
A. Availability of home-based community aged care services	0.143
A1 Availability	0.523
A11 Types of nursing/home-based elderly care services provided by the community	0.383
A12 Number of childcare/home-based elderly care services provided by the community	0.138
A13 The ratio of the number of nursing staff in household/nursing care services to the number of elderly people receiving services	0.094
A14 Number of equipment and facilities in community elderly care services	0.101
A15 The number of community elderly care service sites in the administrative area	0.094
A16 Percentage of certified nursing staff in household/nursing care services	0.101
A17 Number of beds for home-based care services in the administrative area	0.090
A2 Accessibility	0.130
A21 The distance from the residence of the elderly to the home-based elderly care service station	0.429
A22 Convenience of the elderly’s residence to the home-based elderly care service station	0.428
A23 Accuracy rate of nursing staff’s home service time	0.143
A3 Affordability	0.116
A31 Affordability of access to living care services	0.465
A32 Affordability of access to health care services	0.309
A33 Affordability of access to health management services	0.075
A34 Affordability of access to spiritual comfort services	0.076
A35 Affordability of access to cultural and recreational services	0.076
A4 Acceptability	0.115
A41 Acceptance of the content of household/nursing care services	0.500
A42 Acceptance of the household/nursing care model	0.500
A5 Adaptability	0.116
A51 Satisfaction with life care services provided by the community	0.137
A52 Satisfaction with health care services provided by the community	0.201
A53 Satisfaction with health management services provided by the community	0.091
A54 Satisfaction with spiritual comfort services provided by the community	0.088
A55 Satisfaction with cultural and recreational services provided by the community	0.172
A56 Satisfaction with the quality of elderly care services provided by the community	0.143
A57 Satisfaction with the environment in which the community-provided nursing care services are located	0.168
B. Institutional Aged Care Service Accessibility	0.429
B1 Availability	0.527
B11 Types of aged care services provided by institutions	0.097
B12 Number of elderly care services provided by institutions	0.094
B13 Number of facilities for elderly care services in institutions	0.161
B14 Ratio of nursing staff in institutions to seniors served	0.281
B15 Number of elderly care institutions in the administrative area of the district	0.097
B16 Credentialing rate of institutional nursing staff	0.135
B17 Number of beds in elderly care institutions in the administrative region	0.135
B2 Accessibility	0.087
B21 The distance between the residence of the elderly and the nursing facility	0.833
B22 Convenience of the elderly’s residence to nearby elderly care institutions	0.167
B3 Affordability	0.086
B31 Affordability of comprehensive charges for aged care institutions	0.350
B32 Affordability of access to living care services	0.196
B33 Affordability of access to health care services	0.220
B34 Affordability of access to health management services	0.079
B35 Affordability of access to spiritual comfort services	0.079
B36 Affordability of access to cultural and recreational services	0.076
B4 Acceptability	0.086
B41 Acceptance of the content of elderly care services provided by institutions	0.500
B42 Acceptance of institutional pension model	0.500
B5 Adaptability	0.214
B51 Satisfaction with living care services provided by the institution	0.231
B52 Satisfaction with the health care services provided by the institution	0.231
B53 Satisfaction with the health management services provided by the institution	0.053
B54 Satisfaction with spiritual comfort services provided by the institution	0.053
B55 Satisfaction with the cultural and recreational services provided by the institution	0.053
B56 Satisfaction with the quality of elderly care services provided by institutions	0.160
B57 Satisfaction with the surrounding environment where the nursing care services provided by the institution are located	0.110
B58 Satisfaction with the hardware facilities of nursing care services provided by institutions	0.110
C. Administrative services accessibility	0.428
C1 Availability	0.372
C11 The ratio of pension administrators to the elderly population in the administrative area	0.156
C12 The ratio of service managers in elderly care institutions to the number of elderly people receiving services	0.311
C13 The ratio of public pension beds to the number of elderly people in the administrative area	0.104
C14 Average waiting time for admission in public aged care institutions	0.104
C15 The employment rate of senior care administrators in this administrative area with certificates	0.104
C16 The intensity of information disclosure of elderly care services within the administrative region	0.222
C2 Accessibility	0.064
C21 Coverage rate of each sub-district and each community elderly care service center (home elderly care service station, day care center, etc.)	0.750
C22 Coverage of elderly care institutions near the place where the elderly lives	0.250
C3 Affordability	0.172
C31 Proportion of the number of elderly people receiving pension support subsidies in the administrative region	0.442
C32 The total amount of support subsidies for various elderly care services in the administrative region	0.234
C33 The total amount of subsidy received by elderly care institutions in the administrative region	0.168
C34 The total amount of support subsidies received by home-based elderly care service stations in the administrative region (in the past 3 years, including construction subsidies and operating subsidies and operating subsidies)	0.155
C4 Acceptability	0.219
C41 The degree of acceptance of elderly care institutions/home-based elderly care service stations to the administrative functions of the relevant administrative departments	0.325
C42 The degree of acceptance of the supervision and management functions of relevant administrative departments by elderly care home-based elderly care service stations	0.192
C43 The degree of acceptance of the government’s administrative functions to accurately promote the development of elderly care services by elderly care institutions/home-based elderly care service stations	0.242
C44 The degree of acceptance of elderly care institutions/home-based elderly care service stations to the administrative functions of relevant administrative departments to optimize the market environment	0.242
C5 Adaptability	0.173
C51 Old-age care institutions/home-based care service stations for the supervision of old-age services in the administrative region	0.123
C52 Residents’ satisfaction with the elderly care services in the district and county	0.233
C53 Residents’ satisfaction with the administrative department’s old-age service guarantee work	0.189
C54 Satisfaction of residents with pension policies formulated by administrative departments	0.341
C55 Residents’ satisfaction with the work of home-based renovation (indoor) within the administrative area	0.114
